# Ancestry-related assortative mating in Latino populations

**DOI:** 10.1186/gb-2009-10-11-r132

**Published:** 2009-11-20

**Authors:** Neil Risch, Shweta Choudhry, Marc Via, Analabha Basu, Ronnie Sebro, Celeste Eng, Kenneth Beckman, Shannon Thyne, Rocio Chapela, Jose R Rodriguez-Santana, William Rodriguez-Cintron, Pedro C Avila, Elad Ziv, Esteban Gonzalez Burchard

**Affiliations:** 1 Institute for Human Genetics, University of California, San Francisco, 513 Parnassus Ave, San Francisco, CA 94143, USA; 2 Division of Research, Kaiser Permanente, 2000 Broadway, Oakland, CA 94612, USA; 3 Department of Epidemiology and Biostatistics, University of California, San Francisco, 185 Berry Street, San Francisco, CA 94107, USA; 4 Department of Medicine, University of California, San Francisco, 1550 4th St, San Francisco, CA 94143, USA; 5 Biomedical Genomics Center, University of Minnesota, 426 Church St, Minneapolis, MN 55455, USA; 6 Instituto Nacional de Enfermedades Respiratorias, Calzada de Tlalpan 4502, Col. Seccion XVI, CP 14080, Tlalpan, Distrito Federal, Mexico; 7 Centro de Neumologia Pediatrica, CSP, 735 Ave Ponce de Leon, San Juan, 00917 Puerto Rico; 8 Veterans Caribbean Health Care System, 10 Casia St, San Juan, 00921 Puerto Rico; 9 Division of Allergy-Immunology, Feinberg School of Medicine, Northwestern University, 676 N St Clair St, Chicago, IL 60611, USA

## Abstract

**Background:**

While spouse correlations have been documented for numerous traits, no prior studies have assessed assortative mating for genetic ancestry in admixed populations.

**Results:**

Using 104 ancestry informative markers, we examined spouse correlations in genetic ancestry for Mexican spouse pairs recruited from Mexico City and the San Francisco Bay Area, and Puerto Rican spouse pairs recruited from Puerto Rico and New York City. In the Mexican pairs, we found strong spouse correlations for European and Native American ancestry, but no correlation in African ancestry. In the Puerto Rican pairs, we found significant spouse correlations for African ancestry and European ancestry but not Native American ancestry. Correlations were not attributable to variation in socioeconomic status or geographic heterogeneity. Past evidence of spouse correlation was also seen in the strong evidence of linkage disequilibrium between unlinked markers, which was accounted for in regression analysis by ancestral allele frequency difference at the pair of markers (European versus Native American for Mexicans, European versus African for Puerto Ricans). We also observed an excess of homozygosity at individual markers within the spouses, but this provided weaker evidence, as expected, of spouse correlation. Ancestry variance is predicted to decline in each generation, but less so under assortative mating. We used the current observed variances of ancestry to infer even stronger patterns of spouse ancestry correlation in previous generations.

**Conclusions:**

Assortative mating related to genetic ancestry persists in Latino populations to the current day, and has impacted on the genomic structure in these populations.

## Abstract 2

Examination of ancestry-informative genetic markers shows that Puerto Rican and Mexican populations have shown strong assortative mating that continues to this day.

## Background

Mating patterns and preferences have been an active area of research for population geneticists, sociologists, and anthropologists for more than a century. On both a global and local scale, mating does not occur at random. On the larger scale, geographic constraints, such as great distances, high mountains and bodies of water, create local isolation, differentiation and endogamy. The influence of local geography has also been extensively studied [1,2]. However, on a local level, non-geographic factors have greater importance in mate selection. In racially/ethnically heterogeneous societies that characterize the Western hemisphere, race and ethnicity have played a major role in mate selection [3], although inter-racial mating is on the incline. Within racial/ethnic groups and within racially/ethnically homogenous societies, factors such as age, education, occupation, socioeconomic status (SES), height, weight and religious background influence the choice of a mating partner [4-9]. Specific behavioral characteristics are also known to correlate between spouses [10].

Population structure and assortative mating have implications in a wide variety of fields, ranging from genetics to sociology and anthropology. From the perspective of population genetics, the impact depends on the source of the non-random mating. Generally, assortative mating does not affect the frequency of alleles involved with the choice process unless assortment is linked with natural selection or differential reproduction. These are referred to as first moment effects [11]. By contrast, genotype frequencies may be altered by assortative mating, specifically leading to a positive allelic correlation or homozygote excess for loci that are correlated with the mate selection process [4]. These have been referred to as second moment effects [11]. Second moment effects, or correlations, also occur between alleles at different loci, a phenomenon characterized as linkage disequilibrium (LD). Such LD will occur for all pairs of loci that correlate with the source of non-random mating. In the case of multifactorial traits, Crow and Felsenstein [12] have shown that the increase in locus homozygosity is relatively small while the increase in trait variance can be large. The trait variance increase is due primarily to the myriad LD effects among loci.

Assortative mating can also create correlations between previously unrelated traits when these traits are involved in the mating partner selection [4]. These correlations between previously unrelated traits can also have an impact on case-control association studies, significantly increasing type I error rates with loci involved in the assortative mating process [13].

Populations of the Western hemisphere, and particularly Latin America, provide unique opportunities to study population structure and non-random mating, due to the historical confluence of three major racial groups over the past five centuries. Mating among the various migrant and local populations has given rise to new population groups characterized by genetic admixture. During the Spanish colonial period, Spanish colonialists taking Native American or African-descent women as sexual partners was a common practice as early as in the first decades of the 16th century, although social pressure prevented inter-ethnic marriages from becoming widespread [14]. In 1776, the Royal Pragmatic on Marriage was enacted due to 'unequal marriages on account of their size and the diversity of classes and castes of their inhabitants' [15]. The primary purpose of this law was to avoid 'inequality' in the marriage based on an overall assessment not only of skin color, but also of wealth and social status. This 'pigmentocracy' is still observed in some Latin American countries, where the resistance to inter-ethnic marriage is greater among individuals of higher socioeconomic status [3,16].

Within the populations of Latin America, assortative mating has been described to occur based on a variety of factors, including education level, religion, age, family values, anthropometric measurements, and skin pigmentation [16-21]. There has also been debate regarding the degree to which spouse correlations for physical traits such as skin color and anthropometric traits reflect partner selection based on perceived 'race' or selection based on socioeconomic position [16,22], although the two may be confounded in certain settings.

The most significant studies of mating patterns in Latin America have been conducted by Newton Morton and his colleagues in northeastern Brazil [23-25]. These authors studied 1,068 spouse pairs and their offspring of rural origin identified from government records. Subjects were evaluated on an eight-point scale of ancestry based on physical characteristics such as skin pigment, hair color and type, and facial features. The scale reflects the degree of African versus European ancestry. At the same time, the investigators tested 17 blood group and protein markers to genetically estimate African, European and Native American ancestry, within each of the scale categories described above. They found evidence of ancestry correlation between spouses, although they concluded that it was modest [24].

The advent of DNA-based markers now allows us to address the question of non-random mating in Latino populations in a comprehensive way. We use ancestry informative genetic markers (AIMs) to study spouse correlations in two Latino populations, Mexicans and Puerto Ricans. To contrast indigenous versus migrant patterns, we study spouse pairs recruited both from the country/territory of origin (Mexico, Puerto Rico) as well as from the US. We show directly through ancestry estimation that significant spouse correlations in ancestry persist at a high level in all populations, leading to significant LD between unlinked markers, the strength of which is directly related to ancestral allele frequency differences. While both populations show strong assortative mating, the patterns are different, with Mexicans showing spouse correlations in European and Native American ancestry, while Puerto Ricans show spouse correlations in European and African ancestry.

## Results

**Figure 1:**
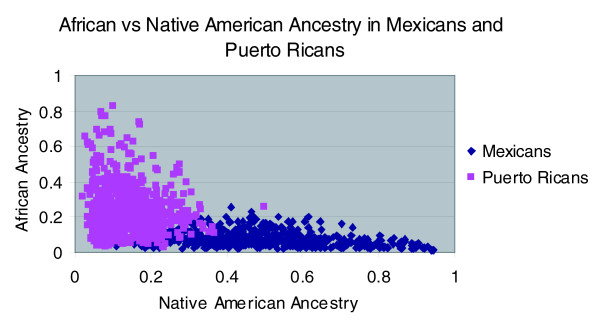
African versus Native American ancestry in Mexicans and Puerto Ricans.

Table 1 provides the average and standard deviation of African, European and Native American ancestry for the wives and husbands, stratified by ethnicity and recruitment site. While both Mexicans and Puerto Ricans have ancestry from all three populations, it is apparent that the Mexicans have predominant European and Native American ancestry but modest African ancestry, while the Puerto Ricans, who also have substantial European ancestry, have greater African ancestry and far less Native American ancestry. Indeed, these studies (and prior ones) indicate that there is only modest overlap in the ancestry distributions for Mexicans and Puerto Ricans (Figure 1). The overlap exists where Native American ancestry ranges from 0.1 to 0.3 and African ancestry from 0 to 0.2. This area of overlap is of particular interest, because it describes individuals who are matched in terms of ancestry but discordant in terms of nationality/ethnicity and culture.

**Table 1:** Mean (standard deviation) ancestries for Latino spouses by recruitment site

			Wives			Husbands		
				
Ethnicity	Site	Number	African	European	Native American	African	European	Native American
Mexican	All	285	0.078 (0.040)	0.437 (0.175)	0.485 (0.180)	0.080 (0.044)	0.425 (0.171)	0.495 (0.180)
	Mexico City	91	0.064 (0.034)	0.322 (0.165)	0.615 (0.172)	0.061 (0.034)	0.322 (0.181)	0.617 (0.194)
	Bay Area	194	0.084 (0.040)	0.491 (0.152)	0.425 (0.150)	0.089 (0.045)	0.473 (0.143)	0.438 (0.142)
								
Puerto Rican	All	377	0.237 (0.141)	0.622 (0.145)	0.141 (0.061)	0.229 (0.127)	0.623 (0.139)	0.148 (0.070)
	Puerto Rico	223	0.232 (0.142)	0.625 (0.148)	0.143 (0.060)	0.223 (0.129)	0.629 (0.140)	0.149 (0.069)
	New York	154	0.244 (0.138)	0.618 (0.141)	0.138 (0.063)	0.238 (0.125)	0.615 (0.138)	0.147 (0.071)

In Mexicans, the predominance of Native American and European ancestry is also reflected in the variances of the three ancestries, where the standard deviation for Native American and European ancestry is large at approximately 0.16, while for African ancestry the standard deviation is much smaller at approximately 0.04. By contrast, in Puerto Ricans, where European and African ancestry are dominant, the variance of African and European ancestry are large (standard deviations approximately 0.14) and the variance of Native American ancestry less (standard deviation 0.065). These variances also have implications for correlations in ancestry within individuals. As expected (Table S1 in Additional data file 1), the correlation between Native American and European ancestry in Mexicans is extremely strong (-0.97). There is also a moderately negative correlation observed between African and Native American ancestry (-0.28). In Puerto Ricans, the correlation between African and European ancestry is strong (-0.89). Because European is the predominant ancestry in the Puerto Ricans, there is also a moderate negative correlation between European and Native American ancestry (-0.35).

Results of *t*-tests comparing average ancestries between spouses, and recruitment site within ethnic group, are given in Table S2 in Additional data file 1. As is apparent in Table 1, there are no significant differences in ancestry between the wives and husbands within any category. There are also no significant differences between the Puerto Ricans recruited from Puerto Rico and those recruited from New York. However, there are substantial ancestry differences between the Mexicans from Mexico City and those from the Bay Area, reflecting a migrant effect. The Bay Area Mexicans have significantly more European and African ancestry and less Native American ancestry compared to the Mexicans from Mexico City (Table S2 in Additional data file 1). This difference may reflect specific geographical or socioeconomic origins of the Mexican migrants to the Bay Area.

To examine a possible role of socioeconomic status on further analyses of these subjects, we examined average ancestries within SES categories for the subset of subjects on whom we had such information (Table S3 in Additional data file 1). Linear regression analysis of ancestry on SES (coded as 1 for low, 2 for moderate, 3 for middle and 4 for upper) was also performed separately for the sexes and ethnicities. There was a non-significant trend towards increased European and decreased Native American ancestry with SES among the Mexican wives but not husbands. However, there was a significant positive relationship of African ancestry with SES and negative relationship of SES with European ancestry among the Puerto Rican wives. SES trends were less clear among the Puerto Rican fathers. We note that because SES was measured based on census-based location information rather than personal information, there may be a loss of sensitivity in these results.

**Figure 2:**
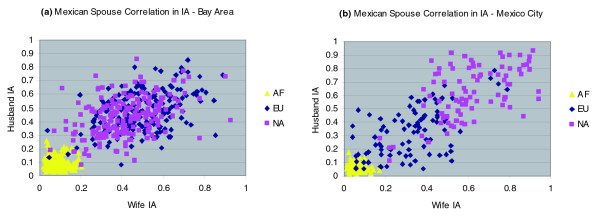
Correlation in individual ancestry for Mexican spouses. Correlation in individual ancestry (IA) for Mexican spouses from **(a) **San Francisco Bay Area and **(b) **Mexico City. AF, African; Eu, European; NA, Native American.

We next examined the between-spouse correlations in ancestry (Table 2). Among the Mexicans, the spouse correlation in European ancestry is extremely high and statistically significant; Native American ancestry shows a similar pattern. By contrast, there is no significant spouse correlation for the African component of ancestry. The correlations for the Mexicans combining the two recruitment sites are confounded by the difference in average ancestries we noted above. However, within site, the spouse correlations for European and Native American ancestry are still high (0.56 to 0.57 for European or Native American ancestry in Mexicans from Mexico City and 0.39 to 0.42 in Mexicans from the Bay Area). Figure 2 depicts the spouse similarity for the three different ancestry components for the two Mexican recruitment sites. Of note, the higher spouse correlation among pairs from Mexico City is due entirely to four couples with particularly high European and low Native American ancestry. Nonetheless, the data show that the spouse ancestry correlation is robust and replicated across the two recruitment sites.

**Table 2:** Between spouse correlations (95% confidence interval) in ancestry, by ethnicity, recruitment site and socioeconomic status

				Ancestry		
				
Ethnicity	Site	SES	Number	African	European	Native American
Mexican	All	All	285	0.068	0.577	0.586
				(-0.048,0.183)	(0.494,0.649)	(0.505,0.658)
	Mexico	All	91	-0.030	0.564	0.568
	City			(-0.234,0.177)	(0.405,0.689)	(0.411,0.693)
	Bay Area	All	194	0.003	0.430	0.392
				(-0.138,0.144)	(0.308,0.538)	(0.266,0.505)
		Low	42	0.131	0.233	0.280
				(-0.180,0.419)	(-0.077,0.501)	(-0.026,0.538)
		Moderate	75	0.048	0.518	0.501
				(-0.181,0.272)	(0.330,0.667)	(0.310,0.654)
		Middle	39	0.268	0.409	0.384
				(-0.052,0.538)	(0.107,0.642)	(0.078,0.624)
						
Puerto Rican	All	All	377	0.328	0.237	0.003
				(0.235,0.416)	(0.139,0.330)	(-0.098,0.104)
	New York	All	154	0.314	0.252	0.146
				(0.164,0.450)	(0.098,0.395)	(-0.012,0.297)
	Puerto	All	223	0.335	0.226	-0.103
	Rico			(0.213,0.446)	(0.097,0.347)	(-0.231,0.029)
		Moderate	56	0.426	0.122	-0.165
				(0.184,0.620)	(-0.146,0.373)	(-0.410,0.102)
		Middle	67	0.281	0.265	0.111
				(0.043,0.488)	(0.026,0.475)	(-0.133,0.342)
		Upper	59	0.458	0.260	-0.361
				(0.229,0.639)	(0.004,0.484)	(-0.564,-0.115)

**Figure 3:**
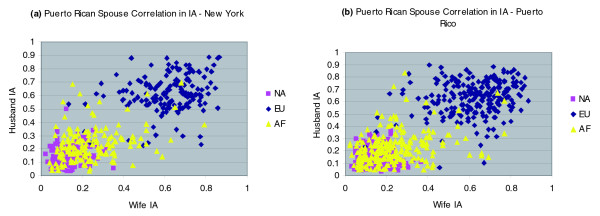
Correlation in individual ancestry for Puerto Rican spouses. Correlation in individual ancestry (IA) for Puerto Rican spouses from **(a) **New York City and **(b) **Puerto Rico. AF, African; Eu, European; NA, Native American.

Within the Puerto Rican spouse pairs, the correlations are high and significant for both European and African ancestry, but not for Native American ancestry. In this case, there are no significant differences in ancestry correlations between the couples from Puerto Rico versus those from New York City. We also note that the spouse correlation in African ancestry (0.33) is somewhat higher than the correlation in European ancestry (0.24), although the difference is not statistically significant. Figure 3 depicts the spouse similarity for Puerto Ricans; the ancestry correlations for Puerto Rican pairs from the two recruitment sites appear quite similar.

An important question is the source of the ancestry correlation between spouses. One possible factor is SES. Therefore, for the Mexicans from the Bay Area and the Puerto Ricans from Puerto Rico, for whom we had such information, we also examined spouse correlations within SES categories (Table 2). The spouse correlations in ancestry persisted within SES categories both in Mexicans and Puerto Ricans, and there was no apparent pattern of increase or decline with SES. As an additional evaluation of the impact of SES, we performed a linear regression analysis, with wife's individual ancestry (IA) as dependent variable and husband's IA and SES as the independent variables. These analyses were performed separately for each of the three ancestry components (Table S4 in Additional data file 1). Here again, we find no attenuation of the significant spouse relationship in European or Native American ancestry in the Mexicans when allowing for SES in the regression model. Similarly, we find no attenuation of African or European ancestry spouse correlation in the Puerto Ricans when including SES in the regression model. SES was not a significant predictor of wife's ancestry in any of the analyses of Mexicans; however, as noted previously, there was a significant positive regression of SES on African ancestry and negative regression of SES on European ancestry among the Puerto Rican wives.

We next evaluated the impact of assortative mating on genotype distributions at individual loci. First, we noted no significant differences in allele frequencies between spouses within recruitment sites, either for the Mexicans or Puerto Ricans (Table S5 in Additional data file 1). However, we did find a large excess of significant allele frequency differences between the Mexican and US recruitment sites for the Mexicans (69% of loci significant at *P *< 0.05). This pattern is consistent with what we previously observed for site-specific ancestry differences for the Mexicans. To determine whether the Mexico City versus Bay Area allele frequency differences were entirely attributable to the ancestry difference between the two sites, we performed a regression analysis of the allele frequency difference chi-square on *δ*_ij_^2^/p*q*, where *δ*_ij _represents the allele frequency difference between ancestral populations i and j, and p* is the allele frequency in the admixed population, q* = 1 - p* (see Materials and methods). The results are given in Table S6 in Additional data file 1. We observed a highly significant regression coefficient for the European-Native American *δ *(0.0339 ± 0.0037), while neither of the other coefficients was statistically significant, nor was the intercept significantly different from 1. Similarly, in an analysis where the intercept term was fixed at 1, the regression coefficients were very close to the unconstrained analysis. Thus, the entire excess of significant allele frequency differences between Mexico City and Bay Area can be attributed to the European-Native American *δ *values at the markers, consistent with the European/Native American ancestry difference between the two sites being the source of site allele frequency differences. As described in Materials and methods, the pairwise sums of regression coefficients provide estimates of the squared difference in ancestry between the two sites. From the regression coefficients in Table S6 in Additional data file 1, we estimate the following ancestry differences between Mexico City and the Bay Area: Native American, √(0.0315 + 0.0025) = 0.184; European, √(0.0315 - 0.0018) = -0.172; African, √(0.0025 - 0.0018) = -0.026. From Table 1, the corresponding numbers are 0.184, -0.160 and -0.024, respectively. Thus, the regression results agree remarkably well with the observed site ancestry differences.

To explore the effect of assortative mating on individual loci, we calculated F values, both for the spouses themselves (within individual correlation) and between spouses (between spouse correlation), as described in Materials and methods. The value F_1 _represents the within spouse allelic correlation, which is derived from the excess of homozygosity among the spouses. The value F_2 _represents the between spouse allelic correlation obtained by sampling one allele from each parent at random, which is also an estimate of the expected value of F_1 _for the children of these spouse pairs (see Materials and methods). Thus, the two values of F allow us to compare the effect of assortative mating across two generations.

The mean values of F_1 _and F_2 _are given in Table 3, stratified by ethnicity and recruitment site. The mean of all F values are significantly greater than 0, although the largest values are observed for F_2 _in Mexicans and F_1 _in Puerto Ricans. For Mexicans, the overall F_1 _and F_2 _values appear reasonably consistent between generations (0.0161 for F_1 _and 0.0172 for F_2_). However, for Puerto Ricans, the overall F values appear higher within spouses (F_1 _of 0.0256) compared to between spouses (F_2 _of 0.0085). This may indicate a decrease in spouse correlation between the generations, but requires additional investigation.

**Table 3:** Mean (standard error) values of allelic correlation within spouses (F_1_) and between spouses (F_2_)

Ethnicity	Site	F_1_	F_2_
Mexican	Mexico City	0.0183 (0.0080)^†^	0.0255 (0.0050)^‡^
	Bay Area	0.0150 (0.0055)^†^	0.0117 (0.0036)^‡^
	All	0.0161 (0.0047)^†^	0.0172 (0.0029)^‡^
			
Puerto Rican	Puerto Rico	0.0268 (0.0047)^‡^	0.0068 (0.0039)*
	New York	0.0239 (0.0060)^‡^	0.0105 (0.0042)*
	All	0.0256 (0.0036)^‡^	0.0085 (0.0030)^†^

*P < 0.05; ^†^*P *< 0.01; ^‡^*P *< 0.001.

We next undertook an analysis to determine the degree to which the significant F values could be attributed to ancestry assortative mating. We did so by linear regression, allowing the F value to be the dependent variable and three independent variables denoted as *δ*_ij_^2^/p*q*, where the i, j subscripts refer to the three possible combinations of the ancestral African, European and Native American populations and p* is the allele frequency in the admixed population (see Materials and methods).

Results are provided in Table 4 (for F_1_) and Table 5 (for F_2_). Among the Mexicans, it appears that the F_1 _values are fully explained by the standardized Native American-European squared delta values of the markers, which were significant for the Bay Area Mexicans and for both groups combined. In these analyses, the intercept term was not different from 0, indicating that the F_1 _distribution was fully explained by the covariate. In the analysis of F_2_, the results were not as clear cut, although again it appears that the Native American-European delta values explain much of the excess. In the analysis including all three delta terms, none were significant in any of the analyses, although the coefficients for the Native American-European delta tended to be largest. However, in analyses including only the Native American-European delta term, this covariate was significant in the analysis of the Bay Area Mexicans and both sites combined. In the final analysis of both groups combined, the intercept term is largely diminished, although still marginally significantly greater than 0.

**Table 4:** Regressions of F_1 _on *δ*^2^/p*q*

		F_1_			
		
Ethnicity	Site	*δ*_AE_^2^/p*q*	*δ*_AN_^2^/p*q*	*δ*_EN_^2^/p*q*	Intercept
Mexican	Mexico City	-0.0052 (0.0049)	0.0066 (0.0047)	0.0187 (0.0110)	-0.0127 (0.0174)
		-0.0047 (0.0048)	0.0054 (0.0043)	0.0125 (0.0069)	0.0 (fixed)
	Bay Area	-0.0028 (0.0039)	0.0056 (0.0037)	0.0274 (0.0086)^‡^	-0.0216 (0.0144)
		-0.0038 (0.0039)	0.0033 (0.0033)	0.0168 (0.0049)^‡^	0.0 (fixed)
	All	-0.0037 (0.0034)	0.0054 (0.0032)	0.0236 (0.0074)^‡^	-0.0160 (0.0124)
		-0.0039 (0.0032)	0.0038 (0.0028)	0.0155 (0.0042)^‡^	0.0 (fixed)
					
Puerto Rican	Puerto Rico	0.0035 (0.0057)	-0.0014 (0.0036)	0.0007 (0.0048)	0.0249 (0.0136)
		0.0110 (0.0041)^†^	0.0015 (0.0032)	0.0054 (0.0041)	0.0 (fixed)
	New York	0.0077 (0.0074)	-0.0074 (0.0045)	0.0028 (0.0061)	0.0289 (0.0175)
		0.0165 (0.0051)^‡^	-0.0040 (0.0041)	0.0082 (0.0052)	0.0 (fixed)
	All	0.0050 (0.0044)	-0.0037 (0.0027)	0.0014 (0.0036)	0.0265 (0.0104)^†^
		0.0131 (0.0031)^‡^	-0.0006 (0.0025)	0.0064 (0.0031)^†^	0.0 (fixed)

In the *δ *subscripts, A represents African, E European and N Native American. Numbers in parentheses are standard errors. ^†^*P *< 0.01; ^‡^*P *< 0.001.

**Table 5:** Regressions of F_2 _on *δ*^2^/p*q*

		F_2_			
		
Ethnicity	Site	*δ*_AE_^2^/p*q*	*δ*_AN_^2^/p*q*	*δ*_EN_^2^/p*q*	Intercept
Mexican	Mexico City	-0.0018 (0.0031)	0.0009 (0.0030)	0.0013 (0.0071)	0.0260 (0.0112)^†^
		-0.0028 (0.0031)	0.0034 (0.0029)	0.0141 (0.0045)^‡^	0.0 (fixed)
	Bay Area	-0.0002 (0.0026)	-0.0024 (0.0025)	0.0075 (0.0057)	0.0123 (0.0096)
		0.0004 (0.0026)	-0.0011 (0.0022)	0.0135 (0.0033)^‡^	0.0 (fixed)
	All	-0.0015 (0.0021)	-0.0012 (0.0020)	0.0039 (0.0046)	0.0202 (0.0078)^†^
		-0.0008 (0.0020)	0.0010 (0.0017)	0.0140 (0.0027)^‡^	0.0 (fixed)
					
Puerto Rican	Puerto Rico	0.0029 (0.0047)	0.0043 (0.0029)	-0.0008 (0.0039)	-0.0063 (0.0111)
		0.0029 (0.0047)	0.0043 (0.0029)	-0.0008 (0.0039)	0.0 (fixed)
	New York	0.0065 (0.0051)	0.0015 (0.0032)	0.0026 (0.0042)	-0.0043 (0.0122)
		0.0052 (0.0035)	0.0010 (0.0028)	0.0018 (0.0035)	0.0 (fixed)
	All	0.0044 (0.0036)	0.0030 (0.0022)	0.0007 (0.0030)	-0.0051 (0.0086)
		0.0028 (0.0025)	0.0024 (0.0020)	-0.0002 (0.0025)	0.0 (fixed)

In the *δ *subscripts, A represents African, E European and N Native American. Numbers in parentheses are standard errors. ^†^*P *< 0.01; ^‡^*P *< 0.001.

Regression analyses on Puerto Rican F_1 _values yielded less clear-cut results. As expected, the largest regression coefficients were for African-European delta terms, although none were formally significant, in the analyses of single sites or for the two sites combined. Also, it appears that the ancestral deltas do not fully explain the excess of homozygosity at these markers. As seen in Tables 4 and 5, the F_2 _values were not as extreme as the F_1 _values, and none of the regression coefficients were significant, although again the largest regression coefficient tended to be for African-European delta terms. After regression, there was no significant intercept term remaining.

As described in Materials and methods, the pairwise sums of regression coefficients provide estimates of the three spouse covariances in ancestry. For the Mexicans we analyzed the two recruitment sites separately, to avoid inflation of spouse covariance due to average ancestry differences between sites. From Table 4, for the regression analysis on F_1 _we estimate the following ancestry covariances for Mexico City: Native American, 0.0125 + 0.0054 = 0.0179; European, 0.0125 - 0.0047 = 0.0078; African, 0.0054 - 0.0047 = 0.0007. For the regression analysis on F_2_, the corresponding covariance estimates are: Native American, 0.0141 + 0.0034 = 0.0175; European, 0.0141 - 0.0028 = 0.0113; African, 0.0034 - 0.0028 = 0.0006. The corresponding observed spouse covariances in ancestry derived from Tables 1 and 2 for Mexico City are: Native American, 0.0190; European, 0.0168; African, -0.0001. Thus, the regression-based estimates for Native American ancestry spouse covariance are quite close to the observed, but the regression-based estimate for European ancestry covariance is somewhat below the observed. For the Bay Area Mexicans, the regression-based covariance estimates for F_1 _are: Native American, 0.0168 + 0.0033 = 0.0201; European, 0.0168 - 0.0038 = 0.0130; African, 0.0033 - 0.0038 = -0.0005. For the corresponding regression analysis on F_2_, we estimate: Native American, 0.0135 - 0.0011 = 0.0124; European, 0.0135 + 0.0004 = 0.0139; African, 0.0004 - 0.0011 = -0.0007. The corresponding observed spouse covariances for Bay Area Mexicans are: Native American, 0.0083; European, 0.0093; African, 0. Here the regression-based estimates appear to somewhat overestimate the actual covariances for Native American and European ancestry. All analyses regarding covariances for African ancestry are consistent in showing no evidence of correlation.

We repeated the same analysis in the Puerto Ricans, but for the two recruitment sites combined. From Table 4, for the regression analysis on F_1 _we estimated the following ancestry covariances: African, 0.0131 - 0.0006 = 0.0125; European, 0.0131 + 0.0064 = 0.0195; Native American, 0.0064 - 0.0006 = 0.0058. For the regression analysis on F_2_, the corresponding covariance estimates are: African, 0.0028 + 0.0024 = 0.0052; European, 0.0028 - 0.0002 = 0.0026; Native American, 0.0024 - 0.0002 = 0.0022. The corresponding observed spouse covariances in ancestry from Tables 1 and 2 for Puerto Ricans are: African, 0.0059; European, 0.0048; Native American, 0. The F_2 _regression-based estimates of spouse covariance for African and European ancestry are comparable to the observed (with a somewhat underestimated European ancestry correlation), while the F_1 _regression-based estimates are higher. This suggests (as does the overall higher mean value for F_1 _than F_2_) that the assortative mating in Puerto Ricans was stronger in the prior generation than in the current one.

To determine whether the excess average F_1 _and F_2 _values might be attributable to specific genomic locations, we created a Q-Q (quantile-quantile) plot of regression residuals against a normal distribution (Figure S1a for Mexicans and S1b for Puerto Ricans in Additional data file 2). In both figures the observed distributions match closely to the expected. Hence, the homozygote excess appears to be a global phenomenon.

Results of the inter-locus (LD) analysis were strikingly different from the single locus analyses. A clear excess of significant chi-square tests was observed in each ethnic group and recruitment site (Table 6). Approximately 15% of tests were found to be significant at the 5% level of significance. Regression analyses of the standardized squared-delta products (for each of the two marker loci involved) were quite revealing (Table S7 in Additional data file 1). For the Mexicans, the European-Native American standardized delta products were extremely predictive of the chi-square, in contrast to the two other delta product covariates. After regression, the intercept terms were greatly attenuated from the corresponding mean chi-squares in Table 6, although still significantly greater than 1. The Puerto Ricans showed a similar pattern, except that the highly significant covariate term in this case was for the African-European squared delta product term (Table S7 in Additional data file 1). As for the Mexicans, the intercept terms were greatly diminished from the corresponding mean values in Table 6, although still somewhat greater than 1. These results show that the primary driver of LD between unlinked loci in this population is ancestral delta values - between Europeans and Native Americans for the Mexicans, and between Africans and Europeans for the Puerto Ricans.

**Table 6:** Chi-square tests of linkage disequilibrium between pairs of markers for spouses combined

Ethnicity	Site	Mean chi-square*	% (N) with *P *< 0.05
Mexican	Mexico City	1.80	0.146 (782)
	Bay Area	1.47	0.105 (564)
	All	3.27	0.152 (814)
			
Puerto Rican	Puerto Rico	1.95	0.161 (861)
	New York	1.43	0.100 (534)
	All	3.38	0.173 (925)

*Chi-square for single sites has 1 degree of freedom (df), expected value is 1.0; for two sites combined, df = 2 and expected value is 2.0.

To search for possible regions with excess LD, we performed another regression analysis, this time on the LD parameter D as a function of the unstandardized delta products (Table 7). As seen previously for the regression analysis of chi-square, the European-Native American deltas were highly significant for the Mexicans, while the African-European deltas were highly predictive for the Puerto Ricans. We then examined the distribution of residuals from the regression by creating a Q-Q plot against a normal distribution (Figure S2 in Additional data file 2). While the overall fit to a normal distribution appears good for both the Mexicans and Puerto Ricans, there do appear to be a few possible outlier points on both ends. The marker pairs involved in the most extreme points (with Z scores greater than +4 or less than -4) are given in Table S8 in Additional data file 1. The most extreme point occurred in Mexicans (Z = +5.09) for markers on chromosomes 2p and 3p. We note that the same pair of markers gave a Z score of +1.10 in the Puerto Ricans. The marker pair on chromosomes 1p and 2q, which gave a Z score of -4.08 in Mexicans, also had a nominally significant Z score in Puerto Ricans (-2.40), while the pair on chromosomes 1p and 17p (Z score of -4.09 in Mexicans) also had a nominally significant Z score in Puerto Ricans, but in the opposite direction (Z = +2.42).

**Table 7:** Regression of linkage disequilibrium parameter D on *δ*_1_*δ*_2_

		*δ *for:			
			
Ethnicity	Site	African-European	African-Native American	European-Native American	Intercept
Mexican	Mexico City	-0.0042 (0.0009)*	0.0044 (0.0006)*	0.0519 (0.0010)^†^	-0.0003 (0.0002)
	Bay Area	0.0004 (0.0007)	0.0006 (0.0004)	0.0322 (0.0007)^†^	0.0002 (0.0001)
	All	-0.0011 (0.0006)	0.0018 (0.0003)*	0.0385 (0.0006)^†^	0.000 (0.0001)
					
Puerto Rican	Puerto Rico	0.0298 (0.0006)^†^	0.0016 (0.0004)*	0.0010 (0.0007)	0.000 (0.0001)
	New York	0.0260 (0.0008)^†^	0.0020 (0.0005)*	0.0009 (0.0009)	0.0001 (0.0002)
	All	0.0283 (0.0005)^†^	0.0018 (0.0003)*	0.0010 (0.0005)*	0.000 (0.0001)

Numbers in parentheses are standard errors. *P < 0.01; ^†^*P *< 0.0001.

**Figure 4:**
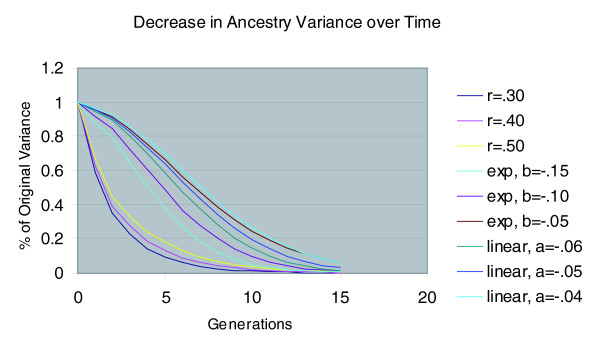
Decay in ancestry variance over time for three spouse correlation models.

We next projected the reduction in ancestry variance over time (see Materials and methods). The results are shown in Figure 4, where we have plotted the proportion of original variance, V_t_/V_0 _against generation. For a constant spouse correlation over time, the variance decreases most rapidly, and is around 10% of its original value after just five generations (for c = 0.3, corresponding to Puerto Ricans) or seven generations (for c = 0.4, corresponding to Mexicans). By contrast, for the linear model (c = 1-at), and the exponential model (c = e^-bt^), the rate of decline of V is slower; a reduction to 10% of the original value occurs between 10 and 13 generations, depending on the model parameters.

To determine the compatibility of the curves in Figure 4 with our own data, we calculated V_t_/V_0 _and r_t _for the current generation of spouses. From the means (*α*) and standard deviations (√V) in Table 1, we derived values of V_t_/V_0 _of approximately 0.11 for European and Native American ancestry in Mexicans and 0.08 for African and European ancestry in Puerto Ricans. By contrast, the proportion of original variance for African ancestry in Mexicans is only 0.02, and for Native American ancestry in Puerto Ricans the value is 0.03. These lower values are consistent with the more modest spouse correlations observed for these ancestry components. All these variance ratios may be slightly inflated due to statistical noise in ancestry estimation. Because there was no correlation of African ancestry in the Mexican spouses, we assumed that the variance observed for African ancestry (0.0016) was primarily due to estimation error, since the actual variance would have decreased rapidly by this point in time. Adjusting the values of V_t_/V_0 _given above for this amount of error variance (an upper bound) reduced the ratios to 0.10 for European and Native American ancestry in Mexicans, and 0.07 for African and European ancestry in Puerto Ricans.

To estimate r_t_, we need to project the value of the LD parameter D to marker loci that are completely informative for ancestry (that is, allele frequency of 1 in one ancestral population and 0 in the other), which corresponds to *δ *values of 1 for both markers. From the regression results presented in Table 7, we can estimate D for *δ *= 1 by simply using the regression coefficient of *δ*_1_*δ*_2_. For Mexicans combined, D = 0.0402. To obtain the value of r_t_, we then need to divide D by *α*(1 - *α*), because *α *and 1 - *α *correspond to the allele frequencies for a marker that is completely informative for ancestry (*δ *= 1). Using the mean ancestry values of Table 1 as *α*, we derive an approximate r_t _value of 0.16. For Puerto Ricans, the value of D is 0.0283; dividing by *α*(1 - *α*), we obtain a value of 0.12. We can rearrange the formula for V_t _given in Materials and methods to V_t_/V_0 _= r_t_/(2 - c_t_) and c_t _= 2 - r_t_/(V_t_/V_0_). Using the values above for V_t_/V_0 _and r_t_, for Mexicans we obtain c_t _= 2 - 0.16/0.10 = 0.40; for Puerto Ricans we obtain c_t _= 2 - 0.12/0.07 = 0.29. These values are close to the observed spouse correlations in ancestry in Table 2. Referring back to Figure 4, we see that our results are consistent with a model of decreasing spouse ancestry correlation over a period of about 9 to 13 generations for Mexicans and 10 to 14 generations for Puerto Ricans. The same formulas given above can also be adapted for linked markers [26]. The assortative mating we observed is expected to enhance the LD between linked markers to an even greater extent than for unlinked markers.

## Discussion

It is of interest to compare our results to those of prior authors who have studied tri-racial populations of northeastern Brazil. Although Krieger *et al*. [24] studied 17 genetic polymorphisms, they did not estimate ancestry at an individual level, but rather within 7 'racial classes' based on a graded scale from 0 to 8 of physical characteristics. However, based on their compilation of spouse pairs for the 7 categories [24] and their estimates of genetic ancestry within each of these categories, we obtained a spouse correlation of 0.46 for African ancestry and 0.45 for European ancestry. These results are comparable to what we observed among the Puerto Ricans, although the Brazilian correlations are somewhat higher. These spouse correlations are also similar to a correlation between spouses of the scale scores derived based on physical characteristics (0.46). This is not surprising, given the very strong correlation between genetically estimated African (European) ancestry and their eight-point scale (correlation = 0.98).

A more recent study by Azevêdo *et al*. [20] examined subjects from the same region of northeastern Brazil, but only used a five-point observed scale of ancestry without genetic markers. However, the spouse correlation in the five-point scale in their data (correlation = 0.47) is quite comparable to that observed in the earlier study from the same region [24].

An important question relates to the actual trait or traits underlying mate selection leading to the spouse correlation in ancestry in these populations. Ancestry is not directly observed, but estimated from genetic markers. One possibility is social, whereby ancestry is associated with social position, and marriages occur within social strata. However, we found only a modest relationship, at best, between SES and ancestry in our study, and the regression of wife's ancestry on husband's ancestry was undiminished when SES was included in the model. Another possibility is geographic origins. If mates are preferentially chosen locally, an ancestry correlation would be induced if ancestry varies geographically. However, among the Puerto Ricans in our study, we found no significant difference between those from New York City and those from Puerto Rico, and also previously found only modest ancestral variation across recruitment sites in Puerto Rico [27]. Re-examining the geographic variation in ancestry in our Puerto Rican subjects [27], we estimate that a spouse correlation of 6 to 8% in African or European ancestry could be induced by such variation; however, this is far short of what we observed, although geographic ancestry variation could be one modest contributor to the observed spouse correlation, assuming that mating preferentially occurs locally.

Among the Mexicans in our study, we noted greater European and lower Native American ancestry among those recruited in the Bay Area than those recruited in Mexico City. Because of this, combining all Mexicans together did increase somewhat the spouse correlations in ancestry; however, the spouse correlations within recruitment sites were nearly as strong. Thus, it appears that geographic heterogeneity in ancestry alone cannot explain the spouse correlations. Another possibility involves physical characteristics, such as skin pigment, hair texture, eye color, and other physical features. Certainly, these traits are correlated with ancestry and are likely to be factors in mate selection. However, the spouse correlation for these traits must be high and the correlation of these traits with ancestry must also be high to explain the observed ancestry correlations. For example, denote the spouse correlation in ancestry by c, the spouse trait correlation by u, and the ancestry-trait correlation by w; then w = √(c/u). If the spouse trait correlation is 0.6 (a reasonably high value), then for a spouse ancestry correlation of 0.3 (Puerto Ricans), the trait-ancestry correlation is 0.7; for a spouse ancestry correlation of 0.4 (Mexicans), the trait-ancestry correlation is 0.8. Previous studies on assortative mating in Latin American groups have retrieved correlation coefficients of 0.29 to 0.46 for education level, 0.48 for skin reflectance, 0.07 to 0.18 for eye and hair color, and 0.16 to 0.24 for different anthropometric measurements [17,18,21].

We also note that the spouses in our study were parents of children with asthma. However, it is unlikely that this selection process has contributed to the spouse correlation because the correlation of genetic ancestry with asthma is only modest, at best [28]. A final assessment of the degree to which these and/or other physical traits may underlie the spouse ancestral correlations observed here requires assessment of these traits within spouse pairs along with ancestry informative markers.

The number of generations since admixing we derived from models allowing for a decrease in spouse ancestry correlation over time is clearly more consistent with the known demographic history of Mexicans and Puerto Ricans [29], and suggests that ancestry assortative mating was even stronger historically than observed in the most recent generations. Although admixing between the indigenous American, European and African populations started to occur in the centuries after the arrival of Columbus and the subsequent importation of slaves from Africa, continuous and large scale migrations to the Americas from Europe continued through the 17th, 18th and 19th centuries. Similarly, the slave trade from Africa continued through the 18th and 19th centuries. Thus, 9 to 14 generations, which corresponds approximately to 225 to 350 years, appears consistent with the general time frame over which the admixing started to occur in substantial numbers, giving rise to the admixed Mestizo populations of Mexico and Puerto Rico [14,30,31].

## Conclusions

We have shown that mating within contemporary Latino populations does not occur at random with regard to ancestry. While both Mexicans and Puerto Ricans show positive assortative mating for ancestry, the pattern between the two populations is quite different. Among Mexicans, the strongest spouse correlations relate to the proportion of Native American and European ancestry, while amount of African ancestry appears to have little impact on mate choice. This is not surprising, given the modest overall level of African ancestry in this population. By contrast, among Puerto Ricans, the strong assortative mating relates to African and European ancestry, while Native American ancestry appears not to contribute to the correlation. While Native American in this population is the smallest ancestral component on average (14%), it is not dramatically less than the average of African ancestry (23%), yet the spouse correlations for these ancestries is dramatically different. Moreover, we did not find any evidence of ancestry asymmetry in the mating patterns. Some authors have described assortative mating by skin color in Latin American populations but with a male preference for lighter-skinned women [16-20]. In our results, there is no evidence of any directionality in partner choice. Ancestry correlation was observed to be a global phenomenon of the genome and not restricted to a few loci.

Our results also reiterate that ancestry variation in Latino populations can be a strong confounder in genetic association studies [32]. As we have shown above, the amount of LD between unlinked markers is directly related to both the ancestry delta values and the variance in ancestry. Assortative mating in these Latino populations will continue to maintain both the ancestry variance and LD over time. However, the patterns observed in these two Latino populations are quite distinct, reflecting strong LD between markers that differentiate Europeans and Native Americans among the Mexicans, versus strong LD between markers that differentiate Europeans and Africans among the Puerto Ricans. It will be of considerable interest to investigate other Latino populations who have varying degrees of African, European and Native American ancestry.

## Materials and methods

### Subjects

The subjects included in this study are part of the Genetics of Asthma in Latino Americans (GALA) study and have been described previously [33]. Subjects are of Mexican and Puerto Rican ethnicity and are parents of childhood asthma patients. Mexican spouse pairs were recruited from both Mexico City and the San Francisco Bay Area. Puerto Rican spouse pairs were recruited from both New York City and from Puerto Rico. Both spouses self-identified as Mexican and all four parents of the spouse pair were identified as Mexican for the Mexico City and Bay Area recruitment sites. For the New York City and Puerto Rico sites, both spouses self-identified as Puerto Rican, and all four parents of spouses were identified as Puerto Rican. The present analysis included 91 Mexican spouse pairs from Mexico City and 194 spouse pairs from the Bay Area for a total of 285 Mexican spouse pairs; there were 154 Puerto Rican spouse pairs from New York and 223 pairs from Puerto Rico, for a total of 377 Puerto Rican spouse pairs.

All subjects provided written informed consent for blood donation and genotyping. The study protocol was approved by the UCSF Committee on Human Research.

### Assessment of socioeconomic status

We used census tract geocoding of income as the basis for SES characterizations of subjects as previously described [27]. The Federal Financial Institutions Examination Council has provided a geocoding/mapping system for this purpose [34]. Census tracts are characterized as low, moderate, middle or upper based on median family income for that census tract compared to median income of the entire metropolitan area. For Puerto Rican subjects from Puerto Rico, SES was defined in terms of the location of the recruitment center; for Mexican subjects from the Bay Area, SES was defined in terms of home residence location.

### Selection of ancestry informative markers

AIMs were selected as described [35]. In brief, biallelic single nucleotide polymorphisms (SNPs) were chosen from an Affymetrix 100K SNP chip panel that showed large allele frequency differences (*δ *of at least 0.5) between pairs of African, European or Native American populations. For the present analysis 107 markers were selected that were widely spaced across all chromosomes, so as to avoid LD in the ancestral populations. A full list of these markers and corresponding chromosome location has been given [35].

### Genotyping

Marker genotyping was performed at the Functional Genomics Core, Children's Hospital Oakland Research Institute as described previously [35]. Briefly, four multiplex PCR assays containing 28, 27, 26, and 26 SNPs, respectively, were performed, followed by single-base primer extensions using iPLEX enzyme and buffers (Sequenom, San Diego, CA, USA). Primer extension products were measured with the MassARRAY Compact System (Sequenom), and mass spectra analyzed using TYPER software (Sequenom) to generate genotype calls.

Quality control was performed on the genotype calls for all Mexican and Puerto Rican subjects. Genotype call rates were generally high and reproducible. The average call rate was 97.6%, and all included markers had a call rate of at least 92%. Three markers were excluded that had call rates below 90% (rs10498919, rs2569029, rs798887), leaving 104 AIMs for subsequent analyses. The final list of markers and their chromosomal locations is given in Table S9 in Additional data file 1.

### Analytic methods

Surrogate ancestral populations were used in this analysis to characterize ancestral allele frequencies for IA estimation. These samples included 37 West Africans, 42 European Americans and 30 Native Americans [35]. We calculated *δ *values between allele frequencies for each pair of ancestral populations for all of the markers. For the African versus European groups, the median *δ *value was 0.56, and 65% of values were greater than 0.30; for the African versus Native American groups, the median *δ *was 0.71, and 83% were greater than 0.30; for the European versus Native American populations, the median *δ *was 0.47, and 59% were greater than 0.30. With this number of markers and distribution of *δ *values, it is predicted that estimated genome-wide IA values are at least 90% correlated with actual values [36].

#### Estimation of ancestry

To estimate individual ancestries, we used the program Structure 2.1 [37,38] using the 104 AIMs described above. Structure was run using the admixture model with unlinked markers, with 50,000 burn-in iterations and 50,000 further iterations. We assumed three ancestral populations, African, European and Native American, and included genotype data on the ancestral populations previously described. The program was run four times, once each for Mexican woman, Mexican men, Puerto Rican women and Puerto Rican men. We analyzed the men and women separately due to possible correlations between spouses. The implementation was similar to what we have done previously [27]. To confirm that the use of three ancestral populations was appropriate, we examined the distribution of LnP(D) for K = 2, 3, 4 and 5. There was a large difference in LnP(D) between K = 2 and K = 3, but not between K = 3 and K = 4 or K = 5. Thus, the optimal value of K for these data was determined to be K = 3. However, this is not surprising as the markers were AIMs and therefore specifically selected to have large allele frequency differences between the three ancestral populations.

#### *t*-tests

Mean ancestries were compared across groups defined by site, gender and SES using *t*-tests.

#### Interclass correlations

Pearson interclass correlations were calculated between ancestries within individuals. Similarly, interclass correlations in ancestry between spouses were calculated. Because means and variances of ancestry were similar in men and women, we also calculated intraclass correlations between spouses. However, these results were virtually identical to the interclass correlations.

#### Single locus analyses

Allele frequency differences between groups were calculated using standard chi-square tests. We tested for Hardy Weinberg equilibrium at marker loci by using the Z-statistic

where n_2 _and n_0 _are the number of homozygotes and n_1 _the number of heterozygotes observed; N = n_2 _+ n_1 _+ n_0_. Under the null hypothesis of no within-locus allelic correlation, Z has a normal distribution with mean 0 and variance 1. We chose to use a one-sided test as opposed to a two-sided chi-square test because we specifically were searching for an excess of homozygotes, as predicted by assortative mating.

Related to Z is the within-locus intraclass allelic correlation F, given by:

Note that Z = F√N. Also, 1 - F represents the proportionate decrease in heterozygosity versus expected under random mating. In future discussion, we refer to this value of F as F_1_, to denote correlation within the first generation (that is, within spouses).

To examine allelic correlations between spouses, we calculated a similar statistic to F. First, we calculated the intraclass correlation *ρ *for the number of 'B' alleles (0, 1 or 2) in the spouse pairs (assume a biallelic locus with alleles B and b). However, because we are correlating two alleles between the spouses, this correlation is not directly comparable to the F value defined within individuals defined above. Hence, to derive a comparable statistic, we created a variable F_2_, defined as the expected intraclass correlation for single alleles selected at random from the two spouses. It can be shown that F_2 _= *ρ *(1 + F_1_)/2. As F_1 _values are generally modest, often F_2 _will be approximately half the intraclass correlation *ρ*.

For comparison, we also calculated interclass correlations for the spouse pairs, which allows for unequal allele frequencies between the two spouses. Because the genotype distributions in wives and husbands were generally extremely similar, the interclass correlations were nearly identical to the intraclass correlations (correlation between correlations ranging from 0.997 to 0.999).

#### Pairwise locus analyses

For pairs of markers, we calculated non-independence of genotype using a likelihood ratio chi-square test, where the double heterozygotes were estimated using maximum likelihood. We also calculated the LD parameter D. Both calculations were performed using the computer package PLINK [39].

#### Linear regressions to estimate effects of ancestry assortative mating

A major goal of this analysis was to examine how genetic structure in Latino populations is influenced by ancestry-related assortative mating. One way to characterize the structure is by examining intra-locus correlations (F statistics) and inter-locus correlations, or correlations between markers (LD parameters r and D). We therefore derived formulas relating the spouse ancestry correlations to expected patterns of allele frequency difference between recruitment sites, F statistics, and D statistics.

First we consider chi-square statistics for allele frequency differences between sites. Let *π*_k _represent the frequency of a marker allele in ancestral population k, where k ranges from 1 to 3, the total number of ancestral populations. Define *δ*_1 _= *π*_1 _- *π*_2_, *δ*_2 _= *π*_1 _- *π*_3 _and *δ*_3 _= *π*_2 _- *π*_3_. Note that *δ*_2 _= *δ*_1 _+ *δ*_3_, so that 2*δ*_1_*δ*_3 _= *δ*_2_^2 ^- *δ*_1_^2 ^- *δ*_3_^2^, a formula we will use later. Further, let *α*_k _represent the proportionate ancestry from population k to the admixed population for the first recruitment site, and *β*_k _represent the proportionate ancestry from population k for the second recruitment site, and let *ε*_k _= *α*_k _- *β*_k_. Note that *ε*_1 _+ *ε*_2 _+ *ε*_3 _= 0. The chi-square statistic for allele frequency difference between site 1 and site 2 is given by:(1)

where:(2)

p_1_' and p_2_' are the allele frequencies in groups 1 and 2, N_1 _and N_2 _are the number of individuals in groups 1 and 2, p* = (N_1_p_1_' + N_2_p_2_')/(N_1 _+ N_2_) and Var represents variance.

Assuming a fixed value for the denominator, we can calculate the expectation (Exp) of the numerator of × ^2 ^in Equation 1 above as:

Dividing this equation by Var(p_1_' - p_2_') gives the approximation:(3)

The numerator in Equation 3 is given by:(4)

Equation 4 shows that Equation 3 for the expectation of *χ*^2 ^can be fit with a linear model in terms of the three covariates, *δ*_i_^2^/Var(p_1_' - p_2_') for i = 1 to 3 via linear regression. If we specify the estimated regression coefficient of *δ*_i_^2^/Var(p_1_' - p_2_') as a_i_, then from the derived regression coefficients we can estimate *ε*_1 _as √(a_1 _+ a_3_), *ε*_3 _as √(a_2 _+ a_3_), and *ε*_2 _= √(a_1 _+ a_2_).

We next consider regression analyses on the statistic F. Recall that F represents the correlation between alleles at a given locus. Consider again a locus with two alleles B and b. Define the binomial random variable S to be 1 if the maternally transmitted allele is B and 0 if b; similarly, define T to be 1 if the paternally transmitted allele is B and 0 if b. Then F can be defined as Cov(S, T)/p*q* where p* is the frequency of B in the combined set of parents and q* = 1 - p* and Cov is covariance. In the analysis of F_1_, p* simply represents the frequency of allele B in the pool of individuals; in the analysis of F_2_, p* represents the frequency of allele B in the pool of spouses combined. Next define the random variable X_i _as the proportionate ancestry from population i in the wife and Y_i _as the proportionate ancestry from population i in the husband, where i ranges from 1 to 3. Note that X_1 _+ X_2 _+ X_3 _= Y_1 _+ Y_2 _+ Y_3 _= 1. Then the random variables S and T can be defined as S = *π*_1_X_1 _+ *π*_2_X_2 _+ *π*_3_X_3 _and T = *π*_1_Y_1 _+ *π*_2_Y_2 _+ *π*_3_Y_3_, respectively. Then, because *π*_2 _is constant, Cov(S, T) = Cov(*π*_1_X_1 _+ *π*_2_X_2 _+ *π*_3_X_3_, *π*_1_Y_1 _+ *π*_2_Y_2 _+ *π*_3_Y_3_) = Cov(*π*_1_X_1 _+ *π*_2_X_2 _+ *π*_3_X_3 _- *π*_2_, *π*_1_Y_1 _+ *π*_2_Y_2 _+ *π*_3_Y_3 _- *π*_2_) = Cov((*π*_1 _- *π*_2_)X_1 _+ (*π*_3 _- *π*_2_)X_3_, (*π*_1 _- *π*_2_)Y_1 _+ (*π*_3 _- *π*_2_)Y_3_) = Cov(*δ*_1_X_1 _- *δ*_3_X_3_, *δ*_1_Y_1 _- *δ*_3_Y_3_) = *δ*_1_^2^Cov(X_1_, Y_1_) + *δ*_3_^2^Cov(X_3_, Y_3_) - 2*δ*_1_*δ*_3_Cov(X_1_, Y_3_), assuming Cov(X_1_, Y_3_) = Cov(X_3_, Y_1_). Now define *κ*_ii _= Cov(X_i_, Y_i_) and *κ*_ij _= Cov(X_i_, Y_j_) for i, j = 1 to 3. Then again noting that *δ*_2 _= *δ*_1 _+ *δ*_3_, we have Cov(S, T) = *δ*_1_^2^*κ*_11 _+ *δ*_3_^2^*κ*_33 _+ (*δ*_1_^2 ^+ *δ*_3_^2 ^- *δ*_2_^2^)*κ*_13 _= (*κ*_11 _+ *κ*_13_)*δ*_1_^2 ^+ (*κ*_33 _+ *κ*_13_)*δ*_3_^2 ^- *κ*_13_*δ*_2_^2^. Therefore, assuming the denominator p*q* is fixed, F is a linear function of the *δ*_i_^2^/p*q*, whose coefficients can be estimated by linear regression. In this case, the coefficients a_i _of *δ*_i_^2^/p*q* are given by a_1 _= *κ*_11 _+ *κ*_13_, a_3 _= *κ*_33 _+ *κ*_13 _and a_2 _= -*κ*_13_. Then note that a_1 _+ a_2 _= *κ*_11_, a_2 _+ a_3 _= *κ*_33_, and a_1 _+ a_3 _= *κ*_11 _+ *κ*_33 _+ 2*κ*_13 _= Cov (X_1 _+ X_3_, Y_1 _+ Y_3_) = Cov(1 - X_2_,1 - Y_2_) = Cov(X_2_, Y_2_) = *κ*_22_. The same linear model and regression coefficients apply to both F_1 _and F_2_, as defined above.

Finally, we consider regression analysis on the LD statistic D. In this case, we examine the co-occurrence of alleles at two loci. Thus, consider loci B_1 _and B_2_, with alleles B_1_, b_1 _at locus B_1 _and B_2_, b_2 _at locus B_2_. Define the random variable S corresponding to locus B_1 _so that S = 1 if allele B_1 _occurs, and 0 if allele b_1_. Define the random variable U similarly for locus B_2_, so that U = 1 if allele B_2 _occurs, and 0 if b_2_. The LD parameter D is defined as Cov(S, U), and χ^2 ^= N [Corr(S, U)]^2 ^where N is the number of individuals and Corr is correlation. Also, Corr(S, U) = Cov(S, U)/[Var(S)Var(U)]^1/2^, Var(S) = p*q*, Var(U) = r*s* where p* is the frequency of B_1_, q* = 1 - p*, r* is the frequency of B_2 _and s* = 1 - r*. Therefore, χ^2 ^= ND^2^/p*q*r*s. For a given individual, assume her(his) three ancestry proportions are represented by the random variables X_i_, where i ranges from 1 to 3. Assume the allele frequency of B_1 _in the three ancestral populations is represented by *π*_i_, for i = 1,3; similarly, the allele frequency of B_2 _in the three ancestral populations is represented by *τ*_i_, for i = 1,3. As before, let *δ*_1 _= *π*_1 _- *π*_2_, *δ*_2 _= *π*_1 _- *π*_3_, and *δ*_3 _= *π*_2 _- *π*_3_. By analogy, we define the ancestral allele frequency differences for the B_2 _locus by φ_1 _= *τ*_1 _- *τ*_2_, φ_2 _= *τ*_1 _- *τ*_3_, and φ_3 _= *τ*_2 _- *τ*_3_. Given the proportions X_i_, D = Cov(S, U) = Cov(*π*_1_X_1 _+ *π*_2_X_2 _+ *π*_3_X_3_, *τ*_1_X_1 _+ *τ*_2_X_2 _+ *τ*_3_X_3_). As before, subtracting the constant *π*_2 _from the first term and *τ*_2 _from the second term, respectively, gives D = Cov((*π*_1 _- *π*_2_)X_1 _+ (*π*_3 _- *π*_2_)X_3_, (*τ*_1 _- *τ*_2_)X_1 _+ (*τ*_3 _- *τ*_2_)X_3_) = Cov(*δ*_1_X_1 _- *δ*_3_X_3_, φ_1_X_1 _- φ_3_X_3_) = *δ*_1_φ_1_Var(X_1_) + *δ*_3_φ_3_Var(X_3_) + (*δ*_1_φ_3 _+ *δ*_3_φ_1_)Cov(X_1_, X_3_). Because Var(X_2_) = Var(1 - X_2_) = Var(X_1 _+ X_3_) = Var(X_1_) + Var(X_3_) + 2Cov(X_1_, X_3_), and *δ*_1_φ_3 _+ *δ*_3_φ_1 _= *δ*_2_φ_2 _- *δ*_1_φ_1 _- *δ*_3_φ_3_, D = *δ*_1_φ_1_Var(X_1_) + *δ*_3_φ_3_Var(X_3_) + (*δ*_2_φ_2 _- *δ*_1_φ_1 _- *δ*_3_φ_3_)(Var(X_2_) - Var(X_1_) - Var(X_3_))/2 = *δ*_1_φ_1_(Var(X_1_) + Var(X_2_) - Var(X_3_))/2 + *δ*_3_φ_3_(Var(X_3_) + Var(X_2_) - Var(X_1_))/2 + *δ*_2_φ_2_(Var(X_1_) + Var(X_3_) - Var(X_2_))/2. In this case, D is a linear function of the *δ*_i_φ_i _for i = 1,3; by linear regression, the coefficients of these terms can be estimated, and are notated as a_i _for i = 1,3. As previously, the regression coefficients can be related to the variances in ancestry by the equations: a1 + a2 = Var(X_2_); a1 + a3 = Var(X_1_); and a2 + a3 = Var(X_3_).

#### Decrease of ancestry variance over time

In theory, the variation in ancestry should decrease from one generation to the next due to recombination between loci. However, the rate of decline will be diminished when there is assortative mating in ancestry. In fact, there is a direct quantitative relationship between the strength of LD between loci, the ancestry variance, and the degree of assortative mating for ancestry over time [26]. Specifically, let c_t _denote the spouse ancestry correlation in generation t, V_t _denote the variance in ancestry at generation t, and r_t _denote the correlation of alleles selected at random at two unlinked loci at generation t (equivalent to the LD parameter r). Let the average ancestry in the population be represented by *α*, which we assume to be unchanged over time. Note that *α*(1 - *α*) represents the variance of ancestry in the generation before admixing first occurred. Then, as shown by Crow and Kimura [26], V_t _= *α*(1 - *α*)r_t_/(2 - c_t_) and r_t+1 _= [r_t _- 1/2_t-1_(r_t _- r_t-1_)]/(2 - c_t-1_). Notice from this formula that when the spouse correlation c is 0, the variance declines by a factor of 1/2 per generation, whereas when c is 1, there is no decline in variance. We iterated the formulas above over 15 generations using 3 different models for the ancestry correlation c: a model where c is constant, a model where c declines linearly over time, and a model where c decreases exponentially over time.

## Abbreviations

AIM: ancestry informative marker; Corr: correlation; Cov: covariance; Exp: expectation; GALA: Genetics of Asthma in Latino Americans; IA: individual ancestry; LD: linkage disequilibrium; Q-Q: quantile-quantile; SES: socioeconomic status; SNP: single nucleotide polymorphism; Var: variance.

## Authors' contributions

NR conceived of the assortative mating study, performed the statistical analyses and drafted the manuscript. SC contributed to the statistical analyses and manuscript writing. MV contributed to the drafting of the manuscript. AB contributed to the data analysis. RS contributed to the analytical theory behind the analyses. CE participated in the genotyping of study subjects. KB oversaw the genotyping of study subjects. ST participated in study subject recruitment. RC participated in subject recruitment and assessments. JRR-S participated in subject recruitment and assessments. WR-C participated in subject recruitment and assessments. PCA participated in subject recruitment and assessments. EZ contributed to the development and analysis of the ancestry informative markers. EGB is the creator of GALA and had overall responsibility for study design and implementation, including subject recruitment and assessment and genotyping, and also contributed to drafting of the manuscript.

## Additional data files

The following additional data files are available with the online version of this paper: supplementary Tables S1 to S9 (Additional data file 1); supplementary Figures S1 and S2 (Additional data file 2).

## Supplementary Material

Additional data file 1 Table S1: within spouse correlations in ancestry. Table S2: *t*-tests of ancestry differences between spouses and between recruitment sites. Table S3: mean (standard deviation) ancestry by socioeconomic status. Table S4: regression of wife's IA on husband's IA and socioeconomic status. Table S5: allele frequency difference chi-square tests between sites and spouses. Table S6: regression of chi-square for Mexico versus US allele frequency difference on *δ*^2^N*/p*q*. Table S7: regression of LD chi-square tests on (*δ*_1_*δ*_2_)^2^/pqrs. Table S8: outlier marker pairs from regressions on D. Table S9: list of ancestry informative markers used in the current study. Click here for file

Additional data file 2 Figure S1: Q-Q plot of residuals from regressions of allelic correlations F_1 _and F_2 _for **(a) **Mexicans and **(b) **Puerto Ricans. Figure S2: Q-Q plot of residuals from regression analysis of the linkage disequilibrium parameter D. Click here for file
