# Supplementary material for: Ancestry-related assortative mating in Latino populations
Source: Genome Biol. 2009 Nov 20;10(11):R132. doi: 10.1186/gb-2009-10-11-r132 (PMC3091325; doi:10.1186/gb-2009-10-11-r132)
Supplement: Additional data file 1 — Table S1: within spouse correlations in ancestry. Table S2: t-tests of ancestry differences between spouses and between recruitment sites. Table S3: mean (standard deviation) ancestry by socioeconomic status. Table S4: regression of wife's IA on husband's IA and socioeconomic status. Table S5: allele frequency difference chi-square tests between sites and spouses. Table S6: regression of chi-square for Mexico versus US allele frequency difference on δ2N*/p*q*. Table S7: regression of LD chi-square tests on (δ1δ2)2/pqrs. Table S8: outlier marker pairs from regressions on D. Table S9: list of ancestry informative markers used in the current study. [file gb-2009-10-11-r132-S1.DOC]

Table S1. Within Spouse Correlations in Ancestry

| Ethnicity | Site | Ancestries | | |
| --- | --- | --- | --- | --- |
|  |  | African-European | African-Native American | European-Native American |
| Mexican | Mexico | -0.196 | -0.094 | -0.958^#^ |
|  | Bay Area | 0.211 | -0.384^#^ | -0.984^#^ |
|  | All | 0.055 | -0.283^#^ | -0.973^#^ |
| Puerto Rican | Puerto Rico | -0.894^#^ | -0.112 | -0.346^#^ |
|  | New York | -0.879^#^ | -0.127 | -0.361^#^ |
|  | All | -0.888^#^ | -0.119 | -0.351^#^ |

^#^P<0.001

Table S2. t-tests of Ancestry Difference between Spouses and between Recruitment Sites

| Ethnicity | Comparison | Ancestry | | |
| --- | --- | --- | --- | --- |
|  |  | African | European | Native American |
| Mexican | Mexico Wives vs Husbands | 0.567 | -0.033 | -0.069 |
|  | Bay Area Wives vs Husbands | -1.114 | 1.209 | -0.900 |
|  | Mexico City vs Bay Area Wives | -4.165^#^ | -8.544^#^ | 9.522^#^ |
|  | Mexico City vs Bay Area Husbands | -5.295^#^ | -7.576^#^ | 8.772^#^ |
| Puerto Rican | Puerto Rico Wives vs Husbands | 0.710 | -0.243 | -0.931 |
|  | New York Wives vs Husbands | 0.386 | 0.240 | -1.266 |
|  | Puerto Rico vs New York Wives | -0.746 | 0.400 | 0.762 |
|  | Puerto Rico vs New York Husbands | -1.074 | 0.908 | 0.132 |

^#^ P<0.0001

Table S3. Mean (s.d.) Ancestry by Socioeconomic Status (SES)

| Ethnicity (Site) | SES | No. | Ancestry | | | | | |
| --- | --- | --- | --- | --- | --- | --- | --- | --- |
|  |  |  | Wives | | | Husbands | | |
|  |  |  | African | European | Native American | African | European | Native American |
| Mexican (Bay Area) | Low | 42 | 0.086 (0.042) | 0.473 (0.134) | 0.441 (0.136) | 0.091 (0.044) | 0.482 (0.128) | 0.427 (0.123) |
|  | Moderate | 75 | 0.086 (0.038) | 0.497 (0.140) | 0.417 (0.136) | 0.084 (0.042) | 0.482 (0.151) | 0.434 (0.147) |
|  | Middle | 39 | 0.080 (0.039) | 0.520 (0.157) | 0.399 (0.146) | 0.087 (0.042) | 0.492 (0.150) | 0.421 (0.148) |
| Puerto Rican (Puerto Rico) | Moderate | 56 | 0.167 (0.110) | 0.684 (0.125) | 0.149 (0.060) | 0.184 (0.098) | 0.676 (0.113) | 0.140 (0.058) |
|  | Middle | 67 | 0.245 (0.151) | 0.606 (0.153) | 0.149 (0.057) | 0.234 (0.139) | 0.600 (0.145) | 0.166 (0.077) |
|  | Upper | 59 | 0.252 (0.129) | 0.608 (0.139) | 0.140 (0.065) | 0.220 (0.137) | 0.638 (0.148) | 0.142 (0.065) |

Note: Regression on SES significant for: African ancestry in Puerto Rican Wives (F=11.55, P<0.001); European ancestry in Puerto Rican Wives (F=8.35, P<0.01).

Table S4. Regression of Wife’s IA on Husband’s IA and Socioeconomic Status

|  | African | | European | | Native American | |
| --- | --- | --- | --- | --- | --- | --- |
|  | IA | SES | IA | SES | IA | SES |
| Mexican | 0.115 (0.074) | -0.003 (0.004) | 0.417 (0.072) ^##^ | 0.021 (0.014) | 0.407 (0.072) ^##^ | -0.018 (0.014) |
| Puerto Rican | 0.396 (0.072) ^##^ | 0.035 (0.012) ^#^ | 0.254 (0.073) ^##^ | -0.033 (0.013) ^#^ | -0.099 (0.066) | -0.004 (0.006) |

Note: Numbers in parentheses are standard errors of regression coefficients;

^#^P<0.01; ^##^P<0.001

Table S5. Allele Frequency Difference Chi-Square Tests Between Sites and Spouses

| Ethnicity | Comparison | Mean Chi-Square | % (N) with P<0.05 |
| --- | --- | --- | --- |
|  |  |  |  |
| Mexican | Mexico City Wives vs Husbands | 0.90 | 0.038 (4) |
|  | Bay Area Wives vs Husbands | 1.14 | 0.067 (7) |
|  | Mexico City vs Bay Area Wives + Husbands | 9.14 | 0.692 (72) |
| Puerto Rican | Puerto Rico Wives vs Husbands | 1.02 | 0.038 (4) |
|  | New York Wives vs Husbands | 0.82 | 0.038 (4) |
|  | Puerto Rico vs New York Wives + Husbands | 0.94 | 0.048 (5) |

Table S6. Regression of Chi-Square for Mexico vs US Allele Frequency Difference on δ^2^N*/ p*q*

| Ancestry Group Delta | | | Intercept |
| --- | --- | --- | --- |
| European-African | Native American-African | Native American-European |  |
| -0.0016 (0.0016) | 0.0030 (0.0015) | 0.0339 (0.0037) ^##^ | -0.147 (1.470) |
| -0.0018 (0.0016) | 0.0025 (0.0014) | 0.0315 (0.0021) ^##^ | 1.0 (fixed) |

Note: Numbers in parentheses are standard errors; ^#^P<0.05; ^##^P<0.0001

Table S7. Regression of LD Chi-Square Tests on (δ_1_δ_2_)2/pqrs

| Ethnicity | Site | Regression Coefficient | | | |
| --- | --- | --- | --- | --- | --- |
|  |  | African-European | African-Native American | European-Native American | Intercept |
| Mexican | Mexico City | -0.0056 (0.0023) | 0.0040 (0.0018) | 0.5646 (0.0250) | 1.209 (0.046) |
|  | Bay Area | 0.0038 (0.0049) | 0.0011 (0.0037) | 0.4612 (0.0261) | 1.054 (0.047) |
|  | All | -0.0040 (0.0023) | 0.0035 (0.0018) | 0.5083 (0.0189) | 1.142 (0.033) |
| Puerto Rican | Puerto Rico | 0.5305 (0.0157) | 0.0192 (0.0066) | -0.0208 (0.0129) | 1.039 (0.059) |
|  | New York | 0.2223 (0.0132) | 0.0029 (0.0053) | -0.0127 (0.0103) | 1.089 (0.048) |
|  | All | 0.3785 (0.0105) | 0.0113 (0.0043) | -0.0169 (0.0085) | 1.062 (0.039) |

Note: Numbers in parentheses are standard errors.

Table S8. Outlier Marker Pairs from Regressions on D

| Ethnicity | Z score | Marker 1 | Chromosome (Location) | Marker 2 | Chromosome (Location) |
| --- | --- | --- | --- | --- | --- |
| Mexicans | -4.44 | rs1517634 | 2 (218 Mb) | rs993314 | 6 (74 Mb) |
|  | -4.09 | rs1934393 | 1 (49 Mb) | rs1990743 | 17 (61 Mb) |
|  | -4.08 | rs10498255 | 1 (102 Mb) | rs1517634 | 2 (218 Mb) |
|  | +5.09 | rs1073319 | 2 (29 Mb) | rs1498991 | 3 (21 Mb) |
| Puerto Ricans | -4.27 | rs2035573 | 3 (130 Mb) | rs1990743 | 17 (60 Mb) |
|  | -4.03 | rs9307613 | 4 (127 Mb) | rs4625554 | 12 (6 Mb) |
|  | +4.22 | rs4762106 | 12 (66 Mb) | rs2208139 | 20 (35 Mb) |

Table S9. List of Ancestry Informative Markers (AIMs) used in the current study.

| dbSNP RS ID Number | Chromosome | Physical Position | Delta_Af_Eu | Delta_Af_NA | Delta_Eu_NA |
| --- | --- | --- | --- | --- | --- |
| rs2817611 | 1 | 11322709 | 0.675 | 0.689 | 0.014 |
| rs6684063 | 1 | 30201812 | 0.611 | 0.056 | 0.667 |
| rs1934393 | 1 | 48578535 | 0.620 | 0.078 | 0.542 |
| rs3768176 | 1 | 56937923 | 0.044 | 0.739 | 0.695 |
| rs3828121 | 1 | 81844793 | 0.131 | 0.700 | 0.569 |
| rs3806218 | 1 | 144518860 | 0.608 | 0.763 | 0.155 |
| rs2592888 | 1 | 156802365 | 0.674 | 0.912 | 0.238 |
| rs4657449 | 1 | 162652658 | 0.012 | 0.650 | 0.662 |
| rs7535375 | 1 | 232954308 | 0.603 | 0.689 | 0.086 |
| rs1073319 | 2 | 29414989 | 0.107 | 0.750 | 0.643 |
| rs1470524 | 2 | 45104050 | 0.563 | 0.311 | 0.252 |
| rs842634 | 2 | 61065756 | 0.234 | 0.739 | 0.505 |
| rs10515919 | 2 | 75515133 | 0.020 | 0.694 | 0.714 |
| rs4852696 | 2 | 83126181 | 0.655 | 0.450 | 0.205 |
| rs3860446 | 2 | 104110751 | 0.615 | 0.028 | 0.643 |
| rs1036543 | 2 | 133886983 | 0.726 | 0.017 | 0.743 |
| rs2711070 | 2 | 159705078 | 0.325 | 0.828 | 0.502 |
| rs868179 | 2 | 177752041 | 0.627 | 0.700 | 0.073 |
| rs10497705 | 2 | 190694557 | 0.120 | 0.749 | 0.629 |
| rs1517634 | 2 | 224386024 | 0.000 | 0.833 | 0.833 |
| rs10498255 | 2 | 231814769 | 0.560 | 0.717 | 0.157 |
| rs304051 | 3 | 4553306 | 0.202 | 0.750 | 0.548 |
| rs1498991 | 3 | 20875097 | 0.032 | 0.694 | 0.726 |
| rs9310888 | 3 | 29261727 | 0.592 | 0.667 | 0.075 |
| rs10510791 | 3 | 57251433 | 0.484 | 0.839 | 0.354 |
| rs1395771 | 3 | 97784481 | 0.632 | 0.090 | 0.543 |
| rs1919550 | 3 | 122685074 | 0.004 | 0.806 | 0.810 |
| rs2035573 | 3 | 132534415 | 0.317 | 0.839 | 0.521 |
| rs1984473 | 3 | 157132197 | 0.575 | 0.911 | 0.336 |
| rs6804094 | 3 | 188378883 | 0.318 | 0.839 | 0.521 |
| rs1398829 | 4 | 21774158 | 0.754 | 0.778 | 0.024 |
| rs719776 | 4 | 33583840 | 0.774 | 0.750 | 0.024 |
| rs10517518 | 4 | 61798796 | 0.087 | 0.811 | 0.724 |
| rs9307613 | 4 | 130816225 | 0.681 | 0.740 | 0.058 |
| rs10519979 | 4 | 150212578 | 0.285 | 0.800 | 0.515 |
| rs10520440 | 4 | 181494895 | 0.214 | 0.833 | 0.619 |
| rs257748 | 5 | 15872353 | 0.425 | 0.161 | 0.586 |
| rs1353251 | 5 | 35902708 | 0.175 | 0.706 | 0.531 |
| rs9292118 | 5 | 55916081 | 0.657 | 0.790 | 0.133 |
| rs6883095 | 5 | 79975120 | 0.313 | 0.828 | 0.514 |
| rs153898 | 5 | 94262695 | 0.655 | 0.050 | 0.705 |
| rs1990745 | 5 | 103458138 | 0.119 | 0.767 | 0.648 |
| rs10515535 | 5 | 143544652 | 0.714 | 0.867 | 0.152 |
| rs1477277 | 5 | 180784684 | 0.655 | 0.700 | 0.045 |
| rs6911727 | 6 | 9061397 | 0.337 | 0.861 | 0.524 |
| rs10484578 | 6 | 35293174 | 0.569 | 0.878 | 0.308 |
| rs993314 | 6 | 73434170 | 0.733 | 0.200 | 0.533 |
| rs9320808 | 6 | 121635172 | 0.766 | 0.106 | 0.871 |
| rs6569792 | 6 | 132675321 | 0.567 | 0.689 | 0.121 |
| rs9295316 | 6 | 158476352 | 0.068 | 0.837 | 0.769 |
| rs10486576 | 7 | 27861415 | 0.044 | 0.744 | 0.700 |
| rs10248051 | 7 | 50861102 | 0.750 | 0.117 | 0.633 |
| rs10214949 | 7 | 78660644 | 0.564 | 0.685 | 0.121 |
| rs10488172 | 7 | 132751390 | 0.187 | 0.772 | 0.586 |
| rs802524 | 7 | 145343383 | 0.651 | 0.689 | 0.038 |
| rs9325872 | 8 | 20490544 | 0.623 | 0.056 | 0.679 |
| rs7463344 | 8 | 33921195 | 0.639 | 0.639 | 0.000 |
| rs4130405 | 8 | 99377358 | 0.190 | 0.800 | 0.610 |
| rs1898280 | 8 | 116031043 | 0.651 | 0.056 | 0.595 |
| rs4733652 | 8 | 129801116 | 0.155 | 0.817 | 0.662 |
| rs2840290 | 9 | 16723957 | 0.593 | 0.039 | 0.632 |
| rs4013967 | 9 | 72354189 | 0.596 | 0.941 | 0.345 |
| rs10491654 | 9 | 97519365 | 0.214 | 0.400 | 0.614 |
| rs10508349 | 10 | 8302970 | 0.044 | 0.711 | 0.755 |
| rs2785279 | 10 | 33713882 | 0.562 | 0.757 | 0.195 |
| rs4934436 | 10 | 90447897 | 0.242 | 0.272 | 0.514 |
| rs1397618 | 10 | 120497262 | 0.658 | 0.706 | 0.048 |
| rs2595456 | 11 | 6849072 | 0.218 | 0.306 | 0.524 |
| rs948360 | 11 | 65882085 | 0.724 | 0.750 | 0.026 |
| rs10501474 | 11 | 80126955 | 0.587 | 0.744 | 0.157 |
| rs567992 | 11 | 105800114 | 0.155 | 0.667 | 0.512 |
| rs879780 | 11 | 129545756 | 0.619 | 0.667 | 0.048 |
| rs4625554 | 12 | 4286565 | 0.131 | 0.700 | 0.569 |
| rs4762106 | 12 | 64304740 | 0.710 | 0.139 | 0.571 |
| rs10506816 | 12 | 78427325 | 0.861 | 0.761 | 0.100 |
| rs249847 | 12 | 97370184 | 0.408 | 0.911 | 0.503 |
| rs4076700 | 12 | 115795273 | 0.627 | 0.694 | 0.067 |
| rs4034627 | 12 | 126750571 | 0.734 | 0.748 | 0.014 |
| rs2585901 | 13 | 19218271 | 0.089 | 0.698 | 0.787 |
| rs10507688 | 13 | 61204229 | 0.167 | 0.733 | 0.567 |
| rs5000507 | 13 | 79886955 | 0.574 | 0.861 | 0.288 |
| rs10492585 | 13 | 103084177 | 0.840 | 0.900 | 0.060 |
| rs9323178 | 14 | 21103774 | 0.276 | 0.790 | 0.514 |
| rs1451928 | 14 | 46330779 | 0.004 | 0.661 | 0.657 |
| rs2296274 | 14 | 59907219 | 0.632 | 0.849 | 0.217 |
| rs10131076 | 14 | 78764426 | 0.623 | 0.694 | 0.071 |
| rs9302185 | 15 | 52670920 | 0.685 | 0.842 | 0.157 |
| rs10520678 | 15 | 86667051 | 0.578 | 0.846 | 0.268 |
| rs30125 | 16 | 14321100 | 0.590 | 0.539 | 0.051 |
| rs1004704 | 16 | 48315382 | 0.187 | 0.839 | 0.652 |
| rs10500505 | 16 | 64718164 | 0.159 | 0.744 | 0.586 |
| rs4130513 | 16 | 78238277 | 0.639 | 0.530 | 0.109 |
| rs10491097 | 17 | 19523240 | 0.592 | 0.078 | 0.514 |
| rs1990743 | 17 | 64494057 | 0.005 | 0.572 | 0.567 |
| rs2253624 | 17 | 70329204 | 0.824 | 0.824 | 0.000 |
| rs1013459 | 18 | 11690534 | 0.667 | 0.733 | 0.067 |
| rs2042762 | 18 | 33529609 | 0.000 | 0.733 | 0.733 |
| rs12953952 | 18 | 65886896 | 0.788 | 0.828 | 0.040 |
| rs888861 | 19 | 40073692 | 0.738 | 0.900 | 0.162 |
| rs708915 | 20 | 8395667 | 0.635 | 0.002 | 0.633 |
| rs2208139 | 20 | 38594383 | 0.167 | 0.800 | 0.633 |
| rs354747 | 20 | 59598070 | 0.560 | 0.683 | 0.124 |
| rs2829454 | 21 | 25194942 | 0.218 | 0.878 | 0.660 |
| rs138022 | 22 | 38856075 | 0.571 | 0.800 | 0.229 |
