# Supplementary material for: Ancestry-related assortative mating in Latino populations
Source: Genome Biol. 2009 Nov 20;10(11):R132. doi: 10.1186/gb-2009-10-11-r132 (PMC3091325; doi:10.1186/gb-2009-10-11-r132)
Supplement: Additional data file 2 — Figure S1: Q-Q plot of residuals from regressions of allelic correlations F1 and F2 for (a) Mexicans and (b) Puerto Ricans. Figure S2: Q-Q plot of residuals from regression analysis of the linkage disequilibrium parameter D. [file gb-2009-10-11-r132-S2.DOC]

Figure S1. Q-Q Plot of Residuals from Regressions of Allelic Correlations F_1_ and F_2_ for (a) Mexicans and (b) Puerto Ricans.

**(a) Q-Q Plot of Residuals of Regressions for F_1_**

**and F_2_ in Mexicans**

-3

-2

-1

0

1

2

3

-4

-2

0

2

4

Expected

F1

F2

**(b) Q-Q Plot of Residuals of Regression for F_1_**

**and F_2_ in Puerto Ricans**

-4

-3

-2

-1

0

1

2

3

4

-4

-2

0

2

4

Expected

F1

F2

Figure S2. Q-Q Plot of Residuals from Regression Analysis of the Linkage Disequilibrium Parameter D.
